# miRNAs Regulation and Its Role as Biomarkers in Endometriosis

**DOI:** 10.3390/ijms17010093

**Published:** 2016-01-13

**Authors:** Josep Marí-Alexandre, Dolors Sánchez-Izquierdo, Juan Gilabert-Estellés, Moisés Barceló-Molina, Aitana Braza-Boïls, Juan Sandoval

**Affiliations:** 1 Unit of Hemostasia, Thrombosis, Atherosclerosis and Vascular Biology, Health Research Institute La Fe, Valencia 46026, Spain; josepmarialexandre@gmail.com (J.M.-A.); moibarcelo@live.com (M.B.-M.); 2 Arrays Unit, Health Research Institute La Fe, Valencia 46026, Spain; Maria.D.Sanchez@uv.es; 3 Maternal Child Area, General Hospital, Valencia 46014, Spain; juangilaeste@yahoo.es; 4 Epigomics Unit, Health Research Institute La Fe, Valencia 46026, Spain

**Keywords:** non-coding RNA, microRNA, biomarker, endometriosis

## Abstract

MicroRNAs (miRNAs) are small non-coding RNAs (18–22 nt) that function as modulators of gene expression. Since their discovery in 1993 in *C. elegans*, our knowledge about their biogenesis, function, and mechanism of action has increased enormously, especially in recent years, with the development of deep-sequencing technologies. New biogenesis pathways and sources of miRNAs are changing our concept about these molecules. The study of the miRNA contribution to pathological states is a field of great interest in research. Different groups have reported the implication of miRNAs in pathologies such as cancer, diabetes, cardiovascular, and gynecological diseases. It is also well-known that miRNAs are present in biofluids (plasma, serum, urine, semen, and menstrual blood) and have been proposed as ideal candidates as disease biomarkers. The goal of this review is to highlight the current knowledge in the field of miRNAs with a special emphasis to their role in endometriosis and the newest investigations addressing the use of miRNAs as biomarkers for this gynecological disease.

## 1. Introduction

Traditionally, in all eukaryotic systems, genes codified proteins, following the central dogma of molecular biology [1] consisting of DNA being transcribed into mRNA and mRNA translated into proteins. The first non-coding RNA (ncRNA) characterized was an alanine tRNA found in baker’s yeast in 1965 [2]. Since then, our understanding of the different and capital roles they play in cells and organisms development and functions has enormously increased. ncRNAs are also assumed to be genomic regulators at different levels and, in case of lower level of sequence conservation, are assumed as evolutionary and biodiversity repositories [3].

Nowadays, it is evident that that RNA is not just a simple messenger between DNA and proteins but growing evidence supports new roles for these molecules, such as regulation of genome organization and gene expression. ncRNAs seem to act at many levels playing important roles in epigenetic processes by controlling differentiation and development and they have been related to very different pathologies (Table 1). Short and long ncRNAs are key regulators of gene expression, genome stability, and defense against foreign genetic elements. ncRNAs are encoded in the genome and never become proteins, demonstrating that the first assumed functions for those molecules have been a lot less than expected [4].

**Table 1:** Classes of non-coding RNAs (ncRNA).

Symbol	Non-Coding RNAs	Functions
tRNA	Transfer RNA	mRNA translation (structural)
rRNA	Ribosomal RNA	mRNA translation (structural)
miRNA	micro RNAs	Post-transcriptional transposon repression
piRNA	Piwi-interacting RNA	DNA methylation, transposon repression
siRNA	Short interfering RNA	RNA interference
snoRNA	Small nucleolar RNAs	RNA modification, rRNA processing
PROMPT’s	Promoter upstream transcripts	Associated with chromatin changes
tiRNAs	Transcripton initation RNAs	Epigenetic regulation
lincRNAs	Long intergenic ncRNA	Epigenetic regulators of transcription
rasiRNA	Repeat associated small interfering RNA	Involved in the RNA interference (RNAi) pathway
eRNA	Enhancer-like ncRNA	Transcriptional gene activation
T-UCRs	Transcribed ultraconserved regions	Regulation of miRNA and mRNA levels
NATs	Natural antisense transcripts	mRNA stability
PALRs	Promoter-associated long RNAs	Chromatin changes
tasiRNA	Trans-acting siRNA	Represses gene expression
lncRNA	Long noncoding RNA	Regulation of gene transcription

## 2. Non-Coding RNA Identification: The ENCODE Project

ncRNAs are RNA fragments that are transcribed from DNA but are not translated into proteins. The main function of ncRNAs is to regulate gene expression at the transcriptional and post-transcriptional level. ncRNAs can be divided into structural and functional regulatory ncRNAs and, at the same time, functional ncRNAs can be subdivided into two main groups according their length; the short ncRNAs (<30 nts) and the long ncRNAs (>200 nts). The amount of ncRNAs codified in the human genome is unknown; however, recent bioinformatic studies have described the sequence of thousands of them [5]. ncRNAs genes include those that are extremely high expressed and showing essential cell functions such as Table 1 describes. A huge contribution to the identification of untranslated sequences has been the ENcyClopedia of DNA Elements (ENCODE http://www.genome.gov/encode/), released in September 2003. The conclusions from this pilot project were published in June 2007 [6]. ENCODE Project was focused on defining RNA transcripts, transcriptional regulator binding sites, and chromatin states in many cell types by different approaches: (a) genomics, to find functional elements where mutations and knock-down models demonstrate the phenotype associated to the genomic sequence; (b) evolutive conservation, as indicator of functional sequences and (c) biochemical approach in models, to characterize ncRNA activity in specific cell type, condition, and molecular processes [7]. Bioinformatic studies on gene regulation and RNA metabolism have described a new variety of functional non-coding sequences, including promoters, enhancers, silencers, insulators, and ncRNA genes. These non-coding elements are associated with chromatin structures or transcription enhancers displaying, for example, histone modifications, DNA methylation, DNase and transcription factor accessibility [8,9,10,11,12,13]. Some of them could be considered at some point as ‘structural sequences’. YRNAs, for instance, are stem loops essential for DNA replication interacting with chromatin and initiation proteins (including the origin recognition complex) [14,15]. Small RNAs are able to modify chromatin structure and to silence transcription by guiding Argonaute-containing complexes to complementary newly transcribed RNAs scaffolds or to gene promoters [16], mediating histone and DNA methyltransferases recruitment. 

## 3. miRNAs

In 1993, a new possibility was included in the genomic and regulatory scheme. Victor Ambros, Rosalind Lee, and Rhonda Feinbaum, during a study of the lin-4 gene controlling the timing of *Caenorhabditiselegans* larval development, described the first miRNA repressing the lin-14 gene [17]. miRNAs are small (21–22 nts) ncRNAs that regulate gene expression and play fundamental regulatory roles in many biological processes [18,19,20]. miRNAs can inhibit the translation of hundreds of mRNAs through sequence specific recognition to the “seed sequence”, and according to the degree of nucleotide compliment, will raise the inhibition of translation and/or degradation of target molecule of mRNAs [18,19,20,21]. Functional analysis of miRNAs have revealed their significant regulatory influence on the expression of target genes involved in both physiological and pathological conditions including gynecological diseases such as endometriosis [22,23,24,25,26,27,28].

## 4. miRNAs Biogenesis

miRNA genes are mainly codified in intergenic or intronic regions of their target genes. In these cases a miRNA gene is transcribed together with its host gene providing a coupled regulation of both. Some pri-miRNA may be codified in the intronic regions of protein and non-protein coding genes or in exons of long non-protein coding RNAs. Consequently, the expression of these miRNAs could be regulated with their host genes [5]. Other miRNA genes show a common promoter forming polycistronic units, containing multiple discrete loops from which mature miRNAs are processed.

**Figure 1:**
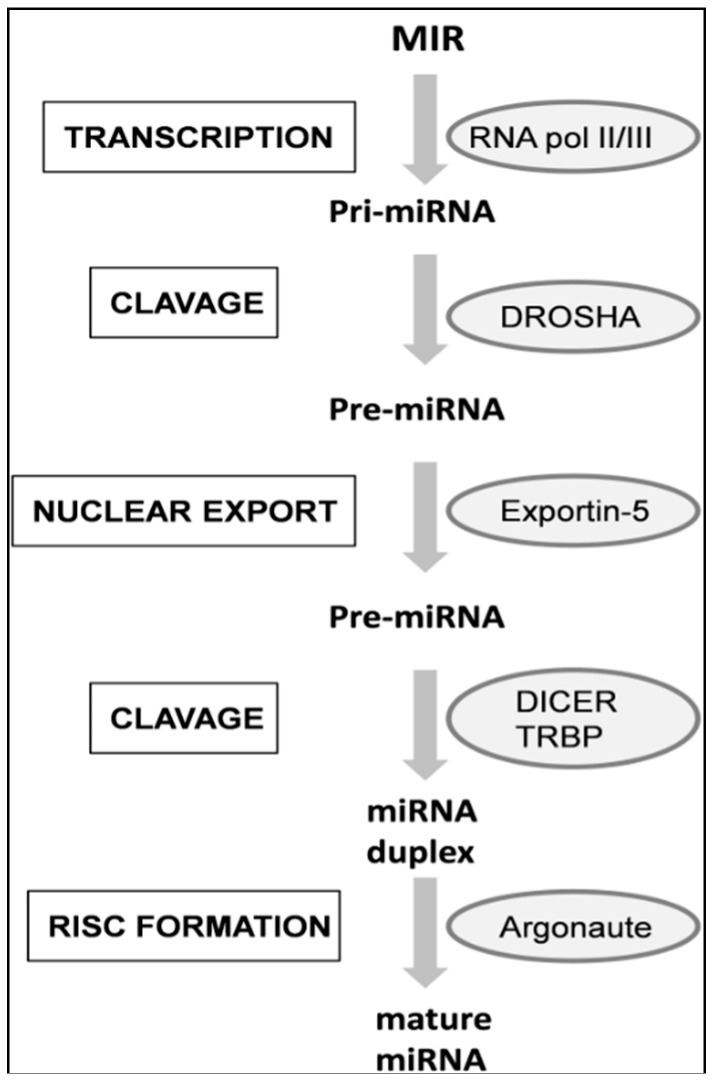
Schematic description of miRNAs biogenesis.

In the canonical miRNA biogenesis pathways described in Figure 1, pri-miRNAs are trimmed by the RNAse III Drosha with the help of a double-stranded RNA binding protein: DGCR8. This protein-complex is known as the Microprocessor and yields ~70 nt stem-loop precursors, termed pre-miRNA, with two nucleotides overhanging at the 3’ and a 5’-phospate. The pre-miRNA is then translocated to the cytoplasm by Exportin-5, a Ran-GTP dependent protein [29,30]. From this point on, further steps are common for miRNAs and exogenous siRNAs.

Once in the cytoplasm, the pre-miRNA is trimmed by Dicer, an RNAse III enzyme, in combination with TRBP (transactivation Response RNA-Binding Protein) [31]. As a consequence, the loop sequence of the hairpin is released and Dicer renders a ~22 nt RNA-duplex with short 3’ overhangs. This step defines the 3’ end of the 5’ strand and the 5’ end of the 3’ strand [18]. Then, Dicer transfers the RNA-duplex to an Ago (Argonaute) protein, which forms the nucleus of RISC (RNA-Induced Silencing Complex). At this time, the complex is called the pre-RISC. The mature RISC is achieved once one of the two strands of the duplex is removed; a process termed “strand selection”.

The main determinant of this process seems to be a thermodynamic factor, mainly determined by the first four nucleotides of the duplex. Hence, the end with weaker interactions will preferentially unwind and remain as the “guide strand”, while the so called “passenger strand” will be discarded [30].

The mature RISC complex is able to scan the cytoplasm searching for mRNA able to pair with the loaded miRNA. The miRNA:mRNA pairing is defined by Watson-Crick interactions between the 3’ UTR (untranslated region) of the mRNA and a short region of nucleotides in positions 2 to 8 of the miRNA known as the “seed sequence”. It is worthy to mention that beyond this general principle of miRNA:mRNA interaction, miRNA pairing with the 5’ UTR has also been defined and observed to be of clinical interest [31] and that additional nucleotides outside the seed sequence can also contribute to determine the mRNA fate [18]. 

It is important to highlight that the DNA sequence is not always the template for the mature miRNA : 6% of human miRNAs suffer RNA editing. In other words, a single pre-miRNA can become multiple mature miRNAs that differ in their length and sequence, named isomiR. The editing process can alter the “seed sequence” conferring different affinity for other targets, modifying the mRNA target selection [5,32]. In this context, the cell-specific expression of different isomiRs implies different protein expression depending on the cell type conferring the biological significance of these miRNA variants. This phenomenon increases the spectrum of miRNA action.

Silencing of mRNAs can be achieved by means of mRNA cleavage or translational repression. Perfect miRNA:mRNA pairing leads to Ago cutting of mRNA approximately at the middle of the miRNA length, whereas imperfect pairing mediates mRNA translational repression by different mechanisms [18].

Apart from the canonical miRNA biogenesis pathway, intronic miRNAs can undergo the “mirtron pathway”. In this non-canoncial biogenesis pathway, the spliced intron renders a lariat in which the 3’ brachpoint is ligated to the 5’ end of the intron. Following the action of Ldbr (lariat debranching enzyme) the lariat is converted in a pre-miRNA that can enter the canonical miRNA biogenesis pathway [5,33]. As the vast majority of intronic miRNAs are found on the sense strand it seems plausible that their expression may be related to that of the host mRNA [34,35] in terms of tissue specificity and relative amount [36].

With the avenue of deep-sequencing strategies, the field of miRNAs research has experienced an unprecedented growth in terms of genetic origins, biosynthetic pathways, and sequence variants [5]. As a result, several ncRNAs have been identified as sources of miRNAs, including snoRNA, lncRNA, and tRNA genes with Drosha- and/or Dicer-independent biogenesis [5].

## 5. miRNAs Nomenclature

The recent advances in high-throughput sequences applied to the miRNA discovery have enormously challenged criteria for miRNA annotation. Nomenclature rules are currently defined by miRBase 21 [37] and the mature form of the miRNA fit the form hsa-miR-XX-3p/5p, where the prefix refers to the species (e.g., hsa- for Homo sapiens). When it is written in capitalized letters, “MIR”, refers to the gene that encodes them; and pre-miRNA and pri-miRNA are named as “mir-”. Distinct precursor sequences and genomic loci expressing identical mature sequences get names of the form hsa-mir-121-1 and hsa-mir-121-2 and adding letters as suffixes denotes mature sequences closely related (hsa-miR-121a and hsa-miR-121b) named miR families. Cloning studies sometimes identify two mature sequences originated from the same pre-miRNA. The ratio between the two opposite mature strands can vary depending on developmental stage, being differentially expressed in distinct tissues or cell types, as well as in pathological conditions [5,30]. Previous nomenclature versions identified the less expressed strand as asterisk * (hsa-miR-XX *). However, recent studies have demonstrated that both strands are functional and the ratio between strands depends on the cellular type or status, the annotation criteria was appropriately changed to the current 5p-/3p-end. Apart from the aforementioned miRNA nomenclature, miRBase also identifies mature miRNAs with a MIMAT accession number. From our own experience, we do recommend authors to refer to studied miRNAs in their manuscripts with the current -3p or -5p suffix and also to include the miRBase MIMAT reference and oligonucleotide sequence in order to avoid future misunderstanding that further nomenclature modifications could introduce. 

In the light of current discoveries in miRNA origins, biosynthetic pathways, and sequence variants, Desvignes and co-workers proposed a revised miRNA nomenclature criterion in the aim of encompassing recent findings in the field. The authors proposed to modify the miRNA nomenclature not only based on biogenesis but also on their function [5].

## 6. Studying miRNAs

**Figure 2:**
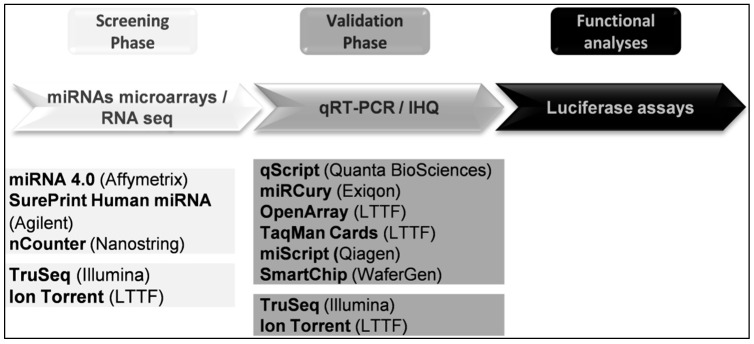
Workflow in a classical study on miRNAs.

In a classic study of miRNAs, the first aim is to assess the miRNA expression profile comparing a pathological group to a control one. The second step in order to corroborate the profiling results is to validate the expression of some of selected miRNAs in a larger cohort of samples. Finally, it would be interesting to prepare functional assays in order to validate the regulation of a specific mRNA translation by the selected miRNA. Summary of technologies applied in miRNA discovering are listed in Figure 2.

The most employed techniques in order to assess miRNA expression profiles are next generation sequencing and microarrays.

### 6.1. RNA Sequencing

Next Generation Sequencing (NGS) was initially developed for sequencing genes faster, cheaper and deeper. The ability to read millions of short fragments of DNA was soon moved into a new methodology, RNAseq, which includes RNA sequencing and quantification. Total RNA from samples is digested and retrotrasncribed through ligation of universal primers. cDNA products are then ligated to primer adaptors for NGS and amplified and isolated in single sequencing reactions on a surface containing millions of ”nanopore” sequencing reactors. Second generation sequencers are able to detect millions of isolated sequencing reactions and to generate data related to nucleotide sequences and the number of reads of each sequence. The bioinformatic software associated with the NGS sequencer is able to align each short RNA fragment with their corresponding genomic region, building a complete transcript read. Analogous to qPCR, the number of reads obtained in RNAseq protocols is correlated to the original amount of cell RNA together with sequence variant detected in the alignment. As limitations, the sensibility of NGS is linked to coverage and throughput, so the presence of over-expressed miRNAs can reduce the ability for detecting miRNAs very low expressed.

### 6.2. miRNA Microarrays

Microarrays have been a revolution in genomic and proteomics fields because of their ability to simultaneously detect thousands of molecules. Thereafter, expression assessment moved from particular genes to whole genomic profiles in which miRNA profiles helped to decode the real complexity of pathological models [38]. Microarray technology is based on the link of millions of genomic fragments on a glass support that are used for sample hybridization (Affymetrix, Agilent, Illumina, NanoString). Samples are fluorescently labeled prior to hybridization. Thus, relative signal on each probe reflects the original amount of miRNA in the studied sample. Hundreds of new ncRNAs are described on an annual basis. For this reason, updating and re-designing of non-coding RNA microarray platforms is mandatory. miRNA expression profiles obtained from microarray platforms should be validated by qPCR in a larger number of samples.

Both techniques have demonstrated their ability in measuring a large number of miRNAs, generating a wide amount of results in the discovery phase. However, nowadays the economic cost is still too expensive to employ them in the validation step. Therefore, quantitative PCR is confirmed to be the most accurate technique to validate results from RNAseq or microarrays

### 6.3. Quantitative PCR or Real-Time PCR

Quantitative PCR (qPCR) or Real-Time PCR (RT-PCR) is a fast, easy, and affordable technique for quantification of miRNAs. Different names depend on applications or thermocycler platforms employed. PCR allows working with very few amount of starting RNA. The first step consists in the synthesis of complementary DNA to miRNA through an adapter following a retrotranscription protocol (RT-PCR). The exponential amplification process in RT-PCR is an extremely sensitive and accurate method for detecting molecules at very low level, thus becoming the gold standard method for quantification of miRNAs in biofluids such as plasma or serum. Few limitations can be attributed to this technique, for example, the limited number of molecules detected depending on plate design (96- or 384-well plate). Pre-amplification and amplification increase sensitivity quantification can be biased in low expressed sequences due to diverse protocol steps. On the other hand, normalization can be difficult because, commonly, “housekeeping” molecules that are used as normalizers can be not stably expressed in some models or pathologies. In any case, so far, qPCR is the reference method for expression validation of other techniques in miRNA research [39]. Nowadays, some high-throughput PCR designed plates are called arrays (Applied, Qiagen, Exiqon). These plates include a customized selection of probes ready-to-use in order to quantify a high number of miRNAs from a single sample reducing technical variations.

### 6.4. In Situ Hybridization (ISH) and Live Cell miRNA Detection

Some companies already specialized in miRNA detection have also developed fluorescent or antibody-conjugated colored-coupled probes for *in situ* miRNA detection (Exiqon, Merk-Millipore). These methodologies allow localizing miRNA molecules in cells or tissues, helping to better characterization of its biogenesis, pathways, and activity. Fluorescent-labeled probes show miRNA localization in fixed or live cells and allow, in some assays, to perform flow sorting of cells expressing concrete regulators [40]. During ISH, a locked nucleic acid (LNA) probe hybridizes to the target sequence at elevated temperature, and then the excess probe is washed away. LNA probes are highly sensitive and have been assayed for therapeutically miRNA antisense therapies [41]. 

## 7. miRNAs as Biomarkers

“Biomarker” definition has been revised by the Biomarker Definitions Working Group in 2001 [42] as “a characteristic that is objectively measured and evaluated as an indication of normal biologic processes, pathogenic processes, or pharmacologic responses to a therapeutic intervention”. In the “omics” era biomarkers can include clinical scoring systems, proteins, gene expression measurements of mRNA, miRNA, DNA, genetic variants of DNA, or metabolites [43].

With the aim to better understand the pathophysiology of different diseases, several authors have reported miRNA expression profiles characteristic of some pathologies (cardiovascular diseases, cancer, diabetes, sepsis, gynecological diseases) [25,26,28,44,45]. The first approach to define a pathological miRNA expression profile is to assess the miRNA expression pattern in the pathological tissue in order to clarify the molecular mechanisms underlying the disease. In this context, miRNAs have reportedly been found in different body fluids, from urine, serum, and plasma to cerebrospinal fluid [46,47].

The presence of miRNAs in different biofluids could be explained by different mechanisms [39,43,48,49]: (a) passive release of miRNAs as consequence of tissue injury, chronic inflammation, cell apoptosis or necrosis, or from cells with a short half-life, such as platelets; (b) active secretion via cell-derived microvesicles including exosomes microparticles and apoptotic bodies [50,51,52]; (c) active secretion by cells in miRNA-protein complexes: High Density Lipoprotein (HDL) and Low Density Lipoprotein (LDL) [53] and Ago2 [54]. Interestingly, Arroyo and co-workers suggested that Ago2-associated miRNAs could be ready to regulate gene expression in recipient cells [54]. Mechanisms (b) and (c) could also offer a rationale for the elevated stability of miRNAs in an RNAse-rich circulation [49]. In light of this evidence, some authors proposed an hormone-like mechanism of action for these miRNAs [48,49] and a role into cell-to-cell communication [55,56,57,58].

Circulating miRNAs, such as the miRNAs released by cancer cells, can bind to Toll-like receptors (TLRs) of immune cells, such as TLR7 in mice or TLR8 in human, to activate NFκB [59,60]. This mechanism could partially explain inflammation related to cancer. 

Importantly, Mitchell and co-workers showed the potential of blood miRNAs as biomarkers for prostate cancer [61], paving the way for further characterizations in other pathologies. In addition, the authors demonstrated that miRNAs were protected from endogenous RNAse activity [61].

## 8. miRNAs Role in Endometriosis

**Figure 3:**
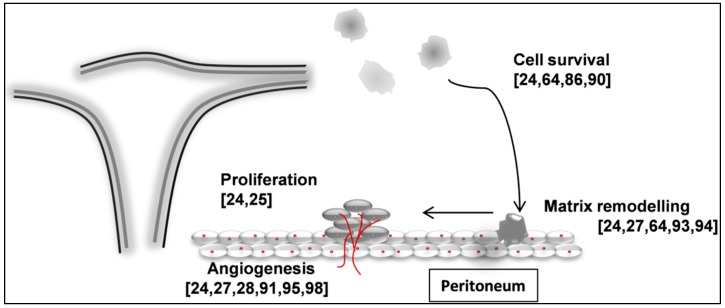
Main systems affected in endometriosis and the references for the most relevant studies about the role of miRNAs regulating them in this disease in square brackets. Red lines represent new blood vessels in formation and red dots cells nuclei.

Different groups, including ours, have studied the potential role of miRNAs in the endometriosis development over years. miRNAs raise as potent regulators of gene expression in the most important systems involved in the pathogenesis of endometriosis. As Figure 3 details, cell survival, matrix remodeling, proliferation, and angiogenesis are essential systems in the pathophysiology of this disease and all of them are potentially regulated by miRNAs [21,22,25,28,62].

## 9. Endometriosis

Endometriosis is a benign estrogen-dependent inflammatory disorder characterized by the presence of endometrial-like tissue outside the uterus. Endometriosis-lesions can be found on the peritoneum (peritoneal lesions), on the ovary either as superficial implants or as endometriotic cysts, and as deeply infiltrative lesions that might extend to the bowel, bladder, and ureter. Pelvic adhesions are often associated with the aforementioned lesions [63]. These lesions are responsible for the main symptoms of endometriosis, pelvic pain and infertility [64]. Whereas innervation at the site of endometriotic lesions is involved in pelvic pain [65], inflammation has been also associated with infertility, what could be explained by a diminished oocyte quality because if their development in an unfavorable environment [66] and also a compromised endometrial receptivity [67].

Endometriosis has been classified as a tumor-like condition by the World Health Organization Histologic Classification of Ovarian Tumors [68]. Indeed, endometriosis shares common features with cancer, as increased local estrogen production, reduced apoptosis, pro-survival, inflammation, tissue invasion, induction of angiogenesis, and dysfunction of immune cells [69]. Since Sampson reported the first case of suspected malignant transformation of ovarian endometriosis [70], several studies have focused on the relationship between endometriosis and gynecological cancers, especially endometrioid and clear cell ovarian carcinoma [71,72,73,74]. However, in a recent review, Guo pointed that existing data is not enough to establish a doubtless causality and highlighted the need for further molecular studies in order to establish an unequivocal phylogenetic relationship between both conditions [69].

Despite its high prevalence and incapacitating symptoms, the exact etiopathogenic mechanism of endometriosis remains unsolved. Burney and Giudice reviewed the theories purposed in recent years with the aim of providing a plausible etiopathogenic mechanism for endometriosis [75]. However, nowadays, the most accepted theory is by far Sampson’s retrograde menstruation proposal, which points that during menstruation, endometrial fragments could migrate through fallopian tubes and reach the peritoneum, being capable to attach, survive, and implant at different locations [70]. It has been demonstrated that all these mechanisms responsible for endometriosis development can be regulated by miRNAs as Figure 3 shows.

## 10. Studying New Biomarkers of Endometriosis

The current gold standard for the diagnosis of endometriosis is laparoscopic examination with histological confirmation of glands and/or stroma in the excised lesions [76]. The need for surgical procedure for diagnosis together with the fear of a cancer diagnosis and the assumption of dysmenorrhea as a normal event could explain the aforementioned delay in time to diagnosis [77]. Taking into account that endometriosis has been reported to be progressive in up to 50% of women [78] and more advanced in women with delayed diagnosis [79] efforts are conducted to achieve a noninvasive diagnosis. In this context, several approaches have been undertaken, such as symptom-based tests [80], or blood tests [81,82,83], but so far neither a non-invasive nor a minimally invasive test has been achieved, remaining as a priority in endometriosis research [84]. Therefore, an ideal test for diagnosis of endometriosis should diagnose patients at initial stages with high sensitivity and specificity.

Due to the anatomical location of this condition, several closely related biofluids have been proposed as a source for noninvasive biomarkers of endometriosis, for instance: urine, plasma/serum, and menstrual blood. In addition, the finding that retrograde menstruation is present in 90% of women but not all of them suffer from endometriosis [67,85] suggests that molecular differences between eutopic endometrium from women with and without endometriosis may exist that lead to the development of the condition in certain women but not in others [67]. As a consequence, if these molecular differences were found to be pathognomonic of the condition they could also provide an opportunity to be considered as biomarkers in biopsied tissues obtained via a minimally invasive procedure.

In the field of miRNAs, differences in miRNA expression between endometriotic lesions and eutopic endometrium from women with endometriosis have been reported [27,86] but few studies have focused in differences between eutopic endometrium from women with and without endometriosis [21,27,87].

Burney *et al.* published one of the first studies addressing the miRNA expression profile in the endometrium of women with and without endometriosis [21]. In this study, miRNA arrays were performed and after qRT-PCR validation, the authors reported a downregulation of four miRNAs (miR-34c-5p, miR-9, miR-9 *, miR-34b *) in the eutopic endometrium from women with endometriosis compared to control endometrium. According to the miRNA regulatory mechanisms, downregulated levels of a miRNA entails the upregulation of its target mRNA translation. Laudanski *et al.* conducted a study enrolling 25 endometriosis-free women and 21 patients with ovarian endometriosis in which the expression of 667 human miRNAs was examined by means of PCR arrays. Validation of the results led to the corroboration that miR-483-5p, a regulator of IGF2, and miR-629-3p, involved in inflammation, were downregulated in the eutopic endometrium of patients in comparison to controls. The authors pointed to the idea that dysregulation of these genes could contribute to the overgrowth of endometrial tissue outside the uterus [87].

Human endometrium is a unique tissue that undergoes complex molecular, cellular, and functional changes on a cyclic basis under ovarian hormone regulation [88,89,90]. These changes are essential for uterine receptivity and can be grouped in three distinct phases: proliferative, secretory, and menstrual [90]. Some authors have described that miRNA expression vary across the menstrual cycle [24]. Particularly, miRNAs targeting several cell cycle regulators were over-expressed in the secretory phase [23]. 

Angiogenesis also plays an important role in the pathogenesis of endometriosis, due that ectopic lesions require neovascularization to proliferate, invade the extracellular matrix and proliferate [27,28,91]. Both Vascular Endothelial Growth Factor A (VEGF-A) and Thrombospondin-1 (TSP-1) represent the most potent pro- and anti-angiogenic factors, respectively, and have been involved in the pathology of endometriosis [92]. Our research group has reportedly observed an increase in the expression of angiogenic and proteolytic factors in endometrial tissues from patients with endometriosis [93,94] and we have suggested that this increase might contribute to the invasive potential of endometrial cells.

The miRNA regulation of angiogenesis has been long reported in several pathologies, including endometriosis [27,28,86,87,95,96]. Two different groups have reported that the angiogenesis regulators, miR-17-5p and miR-20a, are downregulated in the ovarian endometrioma compared to eutopic endometrium [24,87]. The miR-17-92 cluster, also known as oncomir-1, encodes six mature miRNAs (miR-17, miR-18a, miR-19a, miR-19b, miR-20a, and miR-92a) [97]. Recently, our group has reported that the miR-17-92 cluster increases tumour neovascularization by decreasing TSP-1 expression [98]. Therefore, and due to the miRNA mechanism action, a decrease in miR-17-5p levels involve post-transcriptional upregulation of TSP-1 levels. This mechanism may reduce the angiogenic activity in the ovarian endometrioma; therefore, it could explain the low ability in the extracellular matrix invasion of this tissue observed in these ectopic lesions where frequently the ovarian tissue remains preserved.

## 11. Circulating miRNAs as Biomarkers of Endometriosis

The study of circulating miRNAs as biomarkers of endometriosis is an emerging field of research, and to date only few studies have been published both in serum [82] and plasma [81,83].

Wang *et al.* [99] first performed a circulating miRNA array profiling in two pools of sera from 10 patients with endometriosis and 10 control women. After validation of results from array by qRT-PCR in sera from 60 patients and 25 control women, the authors found that miR-199a and miR-122 levels were upregulated and miR-145 *, miR-141 *, miR-542-3p, and miR-9 * downregulated in samples from patients in comparison to control women and could therefore serve as biomarkers of the disease.

Shortly after, another study in plasma was conducted by Jia *et al.* [81]. 23 women with histologically proven endometriosis and 23 endometriosis-free controls were enrolled in the study and a miRNA microarray profiling was performed. Three out of the six miRNAs selected for validation by qRT-PCR (miR-17-5p, miR-20a, and miR-22) were proven to be significantly downregulated in patients and useful to discriminate women with endometriosis from patients.

In 2015, two studies have been published, extending the evidence of miRNAs as putative biomarkers of endometriosis in non-invasive biofluids. In the first case, levels of previously endometriosis-associated miRNAs, miR-135a,b and let-7a-f, were quantified in sera of 24 endometriosis patients and 24 control women. By means of a logistic regression approach, researchers found that combining levels of let-7b, let-7d, and let-7f in the proliferative phase obtained the highest area under the curve value in order to discriminate patients with endometriosis from control women. [82]. Of note, several miRNAs were found to be differently expressed depending on the phase of the menstrual cycle in patients but not in controls, in agreement with previous reports [100]. Finally, Rekker *et al* [83] performed the last published study regarding circulating miRNAs as biomarkers of endometriosis. Based on previous literature, authors selected three miRNAs from the miR-200 family (miR-200a-3p, miR-200b-3p, and miR-141-3p) whose expression was assessed in plasma samples from 61 patients and 65 control women. The expression of all three miRNAs was downregulated in patients and miR-200a-3p and miR-141-3p showed the highest potential as noninvasive biomarkers for this benign condition. Remarkably, authors also analyzed variations of the levels of the three miRNAs of interest with time of sampling (morning/evening) finding lower levels in evening samples, perhaps due to circadian fluctuations in their expression. This is an interesting approach and points to the time of sampling as an important factor to be taken into account when performing circulating miRNAs studies.

## 12. Conclusions

**Figure 4:**
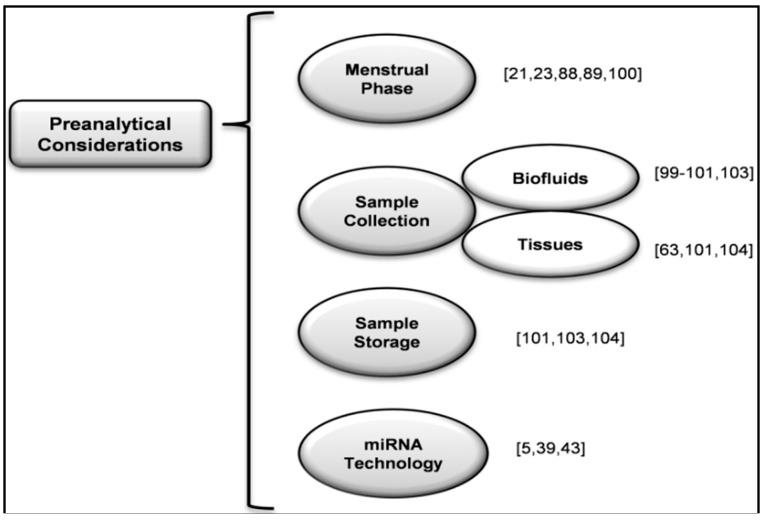
Preanalytical considerations for the miRNA study and relevant literature in square brackets in order to obtain robust conclusions in the standardization of new biomarkers in endometriosis.

Among the epigenetic players, miRNAs have emerged as pivotal post-transcriptional regulators. To do this, these small non-coding RNAs bind to their target mRNAs and inhibit the translation process. The involvement of miRNAs in different pathological conditions has been well established and the miRNA expression profiles have been performed in biopsies from different diseases, including gynecological pathologies as endometriosis. Despite being a benign gynecological pathology, endometriosis deeply impairs the quality of life of affected women in terms of pain and infertility. Nowadays, the gold standard to diagnose endometriosis is laparoscopy. For this reason, several groups including ours are focused on characterizing a non-invasive or semi-invasive biomarker for the diagnosis of endometriosis at initial stages that overcomes the need for the current laparoscopy. Recently, circulating miRNAs have emerged as attractive molecules to be considered as biomarkers [45], although deeper studies are required in order to characterize and validate a miRNA-based diagnostic tool. It is important to highlight the important differences in experimental design and preanalytical protocols among different studies evaluating the same pathology; making it difficult to compare results [40]. For all these reasons, the World Endometriosis Research Foundation (WERF) has published recommendations in order to standardize the data and sample collection, processing and storage [101,102,103,104] and reduce the heterogeneity and improve the reproducibility between studies as summarize the Figure 4. It is essential to unify every step in endometriosis research. The first one is data collection; for this purpose the WERF have elaborated a guide for surgical data collection as well as video/photo of symptom documentation [101,102]. Regarding the study of biofluids, the WERF has defined the protocol in order to standardize the biospecimen collection, processing, and storage [103]. Finally, the collection and storage of tissue samples have also been standardized according to the consensus document [104]. All these documents [101,102,103,104] allow for unifying the studies performed around the world about endometriosis. The aforementioned guides could be also useful for the study and validation in other diseases; these steps avoid the publication of dissimilar studies performed in the “same” disease but employing different protocols. This could be a simple way to obtain robust conclusions and be able to standardize new biomarkers. However, the study of miRNAs as biomarkers implies additional considerations. As it has been previously described, miRNAs are very stable circulating molecules; however heterogeneity among patients seems to be substantial. This feature is even more evident when results from one study are replicated by other group. Based on our own experience, pharmacological treatments, clinical conditions, or even diet can affect severely miRNA expression profiles in plasma.

In conclusion, miRNAs have emerged as new biomarkers valid for diagnostics or prognostics of several diseases. However, standardization in sample and clinical data collection; sample processing and storage; and technical protocols become essential for saving time and money in the assessment of miRNAs as biomarkers.
